# Associations between prefrontal cortex activation and H-reflex modulation during dual task gait

**DOI:** 10.3389/fnhum.2014.00078

**Published:** 2014-02-18

**Authors:** Daan Meester, Emad Al-Yahya, Helen Dawes, Penny Martin-Fagg, Carmen Piñon

**Affiliations:** ^1^Movement Science Group, Department of Sport and Health Sciences, Faculty of Health and Life Sciences, Oxford Brookes University, Headington, OxfordUK; ^2^Department of Physiotherapy, Faculty of Rehabilitation, The University of JordanAmman, Jordan

**Keywords:** gait, dual task, fNIRS, H-reflex, motor control, prefrontal cortex

## Abstract

Walking, although a largely automatic process, is controlled by the cortex and the spinal cord with corrective reflexes modulated through integration of neural signals from central and peripheral inputs at supraspinal level throughout the gait cycle. In this study we used an additional cognitive task to interfere with the automatic processing during walking in order to explore the neural mechanisms involved in healthy young adults. Participants were asked to walk on a treadmill at two speeds, both with and without additional cognitive load. We evaluated the impact of speed and cognitive load by analyzing activity of the prefrontal cortex (PFC) using functional Near-Infrared Spectroscopy (fNIRS) alongside spinal cord reflex activity measured by soleus H-reflex amplitude and gait changes obtained by using an inertial measuring unit. Repeated measures ANOVA revealed that fNIRS Oxy-Hb concentrations significantly increased in the PFC with dual task (walking while performing a cognitive task) compared to a single task (walking only; *p* < 0.05). PFC activity was unaffected by increases of walking speed. H-reflex amplitude and gait variables did not change in response to either dual task or increases in walking speed. When walking under additional cognitive load participants adapted by using greater activity in the PFC, but this adaptation did not detrimentally affect H-reflex amplitude or gait variables. Our findings suggest that in a healthy young population central mechanisms (PFC) are activated in response to cognitive loads but that H-reflex activity and gait performance can successfully be maintained. This study provides insights into the mechanisms behind healthy individuals safely performing dual task walking.

## INTRODUCTION

Walking is a largely automatic process although it is controlled by the cortex, brain stem and spinal cord, and modulated through integration of neural signals from central and peripheral inputs at spinal and supraspinal level (Nielsen, 2003; Yang and Gorassini, 2006). Activation of cortical motor networks, including the motor, premotor, and prefrontal cortex (PFC) has been observed during walking (Fukuyama et al., 1997; Hanakawa et al., 1999). However, whilst it has been reported that cognitive tasks interfere with walking performance (Suzuki et al., 2004; Al-Yahya et al., 2011), the underlying mechanism of how cortical interference affects gait and mobility has not yet been described. Walking has been shown to be facilitated by selective moderation of central drive as a result of inhibitory activity by intracortical neurones which suppress motoneuronal activation (Petersen et al., 2001). This effect is apparent in the strong modulation of the soleus H-reflex throughout the gait cycle whereby the H-reflex decreases or is absent during the swing phase of gait, facilitating ankle dorsiflexion, and increases approaching heel contact and stance phases, thus assisting weight bearing (Yang and Gorassini, 2006; Makihara et al., 2012). The H-reflex is considered to provide valuable information on the involvement of the corticospinal tract in the control of peripheral reflexes and movement during walking (Knikou, 2008a,2008b). There is further evidence of phase-dependent soleus H-reflex modulation, observed during walking in patients with spinal cord injuries (Knikou et al., 2009), which supports the contribution from sensory afferents in walking control. Exploring gait parameters alongside H-reflex and cortical mechanisms may offer an insight into the mechanisms involved in gait control.

In this study we explored the impact of an additional cognitive task, which placed demands on the PFC (McCulloch, 2007), on walking at self-selected and fast walking speeds (Suzuki et al., 2004; Suzuki et al., 2008; Al-Yahya et al., 2009). We set out to investigate PFC activation and any consequential effects on the soleus H-reflex alongside gait performance. To date, a reduced H-reflex amplitude, indicating a depressed spinal excitability to improve stability, has been observed when performing an additional cognitive load during standing (Weaver et al., 2012); but the effect of cognitive load on neural mechanisms during walking has not been explored.

Furthermore, walking speed associated changes have been demonstrated in both central and peripheral mechanisms, where both the activity of the PFC (Suzuki et al., 2004), and H-reflex amplitude were shown to increase with higher walking speed (Simonsen et al., 2012).

We hypothesized that additional cognitive load would increase PFC activity and through projections from the PFC reduce the H-reflex amplitude during normal walking speed. We further expected that increasing both speed and the cognitive load would provoke a further increased activity in the PFC and reduce speed related changes in the H-reflex amplitude (Petersen et al., 2009), with greater changes in the PFC associated with a reduced H-reflex during stance and greater alterations in gait parameters. As such, our study sets out to explore the mechanism behind healthy individuals safely performing dual task walking.

### MATERIALS AND METHODS

Seventeen healthy subjects (7 men; 10 women), 15 right handed and 2 left handed, participated in this study. Mean age was 27.8 ± 6.3 with age range 22–44 years; mean height and weight were 1.75 ± .11 m and 69.1 ± 15.2 kg, respectively. All subjects gave written informed consent according to the Declaration of Helsinki before the start of the experiments and this study was approved by the University Research Ethics Committee. Subjects walked on a treadmill while concurrently performing a cognitive task at a normal and faster walking speed. H-reflexes were elicited in the right soleus and measures of fNIRS were performed on the PFC.

### STUDY DESIGN

Standard methodology, utilizing several practice trials was used to familiarize participants with the treadmill and varying speeds (Woodway ELG 75, Germany) and thus determine preferred walking speed close to normal over ground walking speed (Voloshin, 2000). A faster walking speed was determined by increasing the normal walking speed by 20% (Voloshin, 2000).

The treadmill was programmed for five repetitions of walking and dual task walking alternated with rest periods in which the treadmill was stationary. Both walking and walking with distraction were performed in blocks of 30 s, and rest periods varied from 20 to 40 s. The rest periods had a varying length to prevent subjects anticipating the start of the next block. Subjects performed five repetitions of walking and walking with distraction at each of the two speeds. For the cognitive task, subjects were asked to count backward in steps of seven from a number presented by the investigator.

### fNIRS IMAGING

A continuous wave (782 nm, 859 nm) fNIRS instrument (Oxymon, Artinis Medical Systems, The Netherlands) was used to measure PFC activation. Two identical plastic holders consisting of four optodes each (two sources, two detectors) in a 4-channel arrangement with an inter optode separation of 30 mm were placed on each participant’s forehead using a custom-built spring-loaded array optode holder covering the area linking Fp1, F3, and F7 and the area linking Fp2, F4, and F8 according to the international 10–20 EEG electrode system, which corresponds to the left and the right PFC, respectively (Leff et al., 2008). To monitor hemodynamic responses, blood pressure, and heart rate were measured at baseline and at the end of the program.

### H-REFLEXES

H-reflexes were elicited in the right soleus muscle (SOL) during single and dual task blocks. A constant current high voltage stimulator (Digitimer Ltd. DS7A, UK) was used to elicit H-reflexes and M-waves. H-reflex recruitment curves were obtained while subjects were standing. H_ max_ and M_ max_ were measured to determine the intensity which elicited 20–25% of M_ max_ (Simonsen and Dyhre-Poulsen, 1999; Phadke et al., 2010). A footswitch (Odstock Medical Ltd, UK) under the subject’s right heel provided data to time the stimulation within the gait cycle. The footswitch was used to trigger the stimulator to elicit a H-reflex during mid-stance (30% of gait cycle; Hughes and Jacobs, 1979). To prevent depression of the H-reflex and subject anticipation of the reflex, stimulations were given every four, five, or six heel strikes; corresponding to an inter-stimulus-time (ISI) of 4–5 s, which is known to be long enough to measure consecutive H-reflexes (Knikou and Taglianetti, 2006; Jeon et al., 2007).

### EMG RECORDING AND NERVE STIMULATION

Based on earlier research (Capaday and Stein, 1986,^1987^; Simonsen and Dyhre-Poulsen, 1999), the right SOL was selected for EMG recordings. Ag–AgCl electrodes (55 mm diameter) were placed on the muscle belly and as a stimulating electrode on the tibial nerve (Konrad, 2005). The cathode was placed in the popliteal fossa with the anode at a distance of 2 cm medial to the cathode. Researchers located the nerve using small moveable electrodes, before positioning the actual stimulation electrodes, which were secured with Velcro tape to prevent slippage during locomotion. EMG leads were attached to the leg and upper body to reduce movement artifacts and prevent subjects from tripping.

### STEP TIME

Step time was measured using an inertial measuring unit (Philips, Eindhoven, The Netherlands) comprising a tri-axial accelerometer, gyroscope, and magnetometer placed on the center of mass (Esser et al., 2012). Post-processing and analysis was performed in a pre-written program in LabVIEW2010 (National Instruments, Austin, TX, USA). Step time was taken as the gait variable of interest with the time interval between trough-to-trough center of mass excursions during one gait cycle (Esser et al., 2009).

### DATA PROCESSING

Raw fNIRS signals were collected at a sample rate of 10 Hz. Deoxy-Hb and Oxy-Hb concentrations were calculated (Oxysoft 2.1.6), filtered with a low pass filter set at.67 Hz (Labview 6.1) and visually inspected for motion artifacts, missing signals, and noisy signals. Blocks with missing signals or artifacts were excluded from analysis. A moving average filter with a width of 4 s was used to smooth the signal. Block averages of the 5 task + rest repetitions were calculated and the middle 10 s of each task and rest periods used for statistical analyses. To offset low spatial resolution of fNIRS, and provide a better indication of general measured activity in the PFC, the four channels on both the left PFC and the right PFC were averaged.

Signal software was used for data acquisition and analysis (CED Signal 3.09, UK). EMG signals were pre amplified 1000 times and high passed filtered at 30 Hz (NL844; Digitimer). Consequently signals were low pass filtered at 200 Hz (NL135; Digitimer) before H-reflexes were sampled at 1000 Hz (Tokuno et al., 2007). Peak-to-peak amplitude of the H-reflex measured during walking was normalized by expressing the walking H-reflex as a percentage of the standing H-reflex elicited at the same intensity. Variability of normalized H-reflexes was determined using the standard deviation.

### STATISTICS

Descriptive statistics were performed on demographic and gait control parameters. Paired *t*-tests were used to examine differences in hemoglobin concentrations during task and rest blocks. The effects of task and speed on brain measures, H-reflex amplitudes and step times were examined using repeated measures ANOVA models. To investigate relationships between central and peripheral mechanisms, changes in Oxy-Hb concentrations, H-reflex amplitude variability, and step time variability were explored through Pearson correlations. For all statistical tests, alpha level was set at 0.05 a priori, and SPSS Bonferroni adjusted *p*-values are quoted.

## RESULTS

### DESCRIPTIVES

Individuals’ average self-selected normal walking speed was 1.22 ± SD 0.24 m/s, range 0.7–1.5 m, and faster walking speed was 1.48 ± 0.26 m/s, range 1.0–1.7 m/s. Blood pressure and heart rate were stable with a mean blood pressure of 117 ± 10.6/75 ± 6.7 mmHg, range 98/63 mmHg to 136/89 mmHg and a mean heart rate of 75 ± 12.3 bpm, range 60–109 bpm. Blood pressure and heart rate did not significantly (*p* > 0.05) change from baseline to normal and faster walking speed. Cognitive task score was not significantly (*p* > 0.05) different between speeds. Average answer rate was 10.3 ± 3.8 answers during normal walking and 10.5 ± 3.8 during faster walking with, respectively, mean error rates of 0.4 ± 0.4 and 0.4 ± 0.3.

### NIRS IMAGING

**FIGURE 1:**
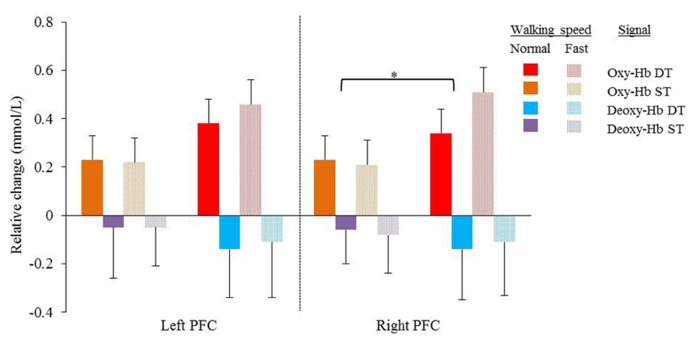
**Mean relative changes and standard deviations in Oxy-Hb (red and orange) and Deoxy-Hb (purple and blue) during normal and fast (dotted bars) walking in the left and right cortex.** Results of single task (orange and purple) and dual task walking (red and blue) are presented. PFC = prefrontal cortex, Oxy-Hb = oxy hemoglobin, Deoxy-Hb = deoxy hemoglobin, ST = single task, DT = dual task. Significant higher Oxy-Hb concentration change during dual task walking compared to single task walking in the right cortex (*p = 0.049).

**FIGURE 2:**
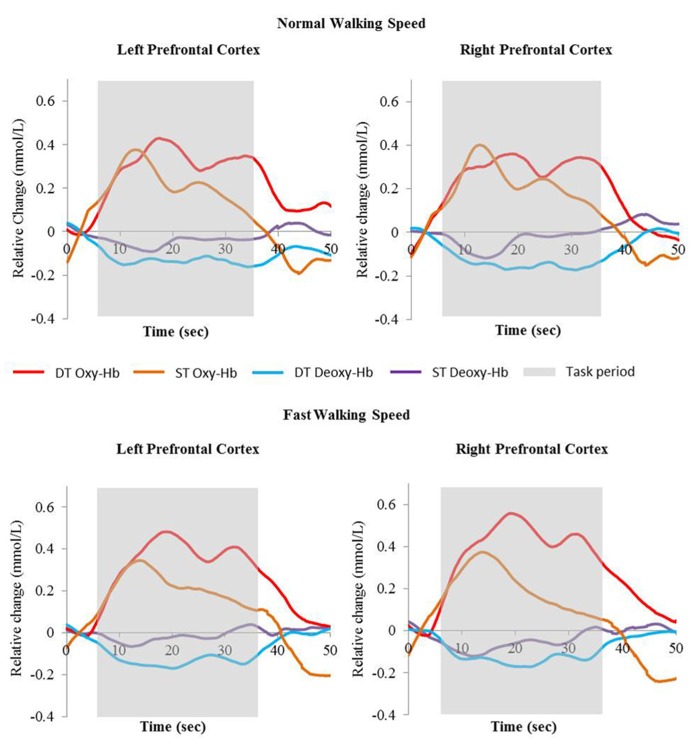
**Relative change of Oxy-Hb (red and orange) and Deoxy-Hb (blue and purple) concentrations during single task walking and dual task walking at normal and fast walking speed.** Task period are indicated in gray. Oxy-Hb = oxyhemoglobin, Deoxy-Hb = deoxyhemoglobin, ST = single task, DT = dual task.

Average Oxy-Hb and Deoxy-Hb concentrations are summarized in **Figures 1** and **2**. Repeated measures ANOVA results are shown in **Table 1**. For single and dual task blocks at normal and faster walking speed, relative Oxy-Hb concentrations were significantly (*p* < 0.05) higher during the task compared to the average rest block followed after each task in both hemispheres. Deoxy-Hb changes were significantly (0.011) lower during dual task blocks compared to rest in the right PFC when walking at a faster walking speed.

**Table 1:** Repeated measures showing the effect of task and speed on Oxy hemoglobin and Deoxy hemoglobin concentrations in the left and right prefrontal cortex.

Summary statistics of ANOVA for Oxy and Deoxy hemoglobin concentrations								
		Left PFC hemisphere				Right PFC hemisphere		
	Oxy-Hb		Deoxy-Hb		Oxy-Hb		Deoxy-Hb	
Effect	*F*	*Sig.*	*F*	*Sig.*	*F*	*Sig.*	*F*	*Sig.*
Task	3.535	0.080	3.396	0.085	4.632	0.049^*^	2.107	0.169
Speed	0.213	0.651	0.188	0.736	1.776	0.204	0.045	0.835
Task*Speed	0.471	0.503	0.076	0.786	2.425	0.142	1.231	0.286

Task = single and dual task walking, speed = normal and faster walking speed, PFC = prefrontal cortex, Oxy-Hb = oxy hemoglobin, Deoxy-Hb = deoxy hemoglobin. Significant higher Oxy-Hb concentration change during dual task walking compared to single task walking in the right prefrontal cortex (*p = 0.049).

In the right cortex Oxy-Hb concentrations increased significantly with dual task (*F* = 4.632; *p* = 0.049) from 0.23 ± 0.1 mmol/l to 0.34 ± 0.1 mmol/l at normal speed and from 0.21 ± 0.1 to 0.51 ± 0.1 at faster speed. In the left cortex, a trend was shown toward significant increases (*F* = 3.535; *p* = 0.080) of Oxy-Hb concentrations with dual task, with increases from 0.23 ± 0.1 mmol/l to 0.38 ± 0.1 mmol/l and 0.22 ± 0.1 mmol/l to 0.46 ± 0.1 mmol/l for normal and faster walking speed, respectively. Deoxy-Hb concentrations were not significantly affected by task or speed. Increases and decreases in Oxy-Hb and Deoxy-Hb were not significantly different between speeds. No significant interactions were found between task and speed for either Oxy-Hb and Deoxy-Hb concentrations.

### H-REFLEX AND STEP TIME

**FIGURE 3:**
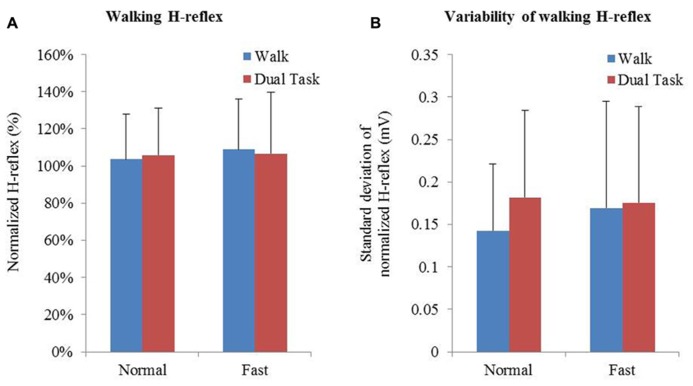
**FIGURE 3.** **(A)** Means and standard deviation of normalized H-reflex. **(B)** Mean variability of H-reflex+standard deviation. Variability of the normalized H-reflex and step time was measured using the standard deviation.

Averages and variability of H-reflex amplitudes and step times are described in **Table 2** and **Figure 3**. Changes of mean normalized H-reflex, step time, and variability of H-reflex and step time were not significantly (*p* > 0.05) different between tasks and walking speeds. Furthermore repeated measures ANOVAs did not show interactions between task and speed for both parameters (see **Table 3**).

**Table 2:** Averages + standard deviations of H-reflex amplitude, variability, step times, and step time variability.

H-reflex and step time averages and variability				
	Normal walking speed		Fast walking speed	
	Single task	Dual task	Single task	Dual task
H-reflex (%)	103.7 ± 24.4	105.9 ± 25.5	109.0 ± 26.9	106.7 ± 33.2
H-reflex variability (%)	14.2 ± 7.8	18.1 ± 10.3	16.9 ± 12.5	17.6 ± 11.3
Step time (ms)	528.4 ± 41.3	532.4 ± 46.1	524.3 ± 39.8	517.6 ± 38.3
Step time variability (ms)	105.0 ± 134.1	124.6 ± 139.4	63.4 ± 81.3	54.1 ± 48.5

*Variability of the normalized H-reflex and step time was measured using the standard deviation.*

**Table 3:** Repeated measures showing the effect of task and speed on normalized H-reflex, H-reflex variability, step time, and step time variability.

Summary statistics of ANOVA for H-reflex amplitudes and step times								
	H-reflex		H-reflex variability		Step time		Step time variability	
Effect	*F*	*Sig.*	*F*	*Sig.*	*F*	*Sig.*	*F*	*Sig.*
Task	0.001	0.973	2.266	0.153	0.966	0.341	1.436	0.251
Speed	0.868	0.366	0.376	0.549	0.108	0.746	0.205	0.658
Task*Speed	0.951	0.345	2.255	0.154	3.339	0.088	3.387	0.087

*Variability of the normalized H-reflex and step time was measured using the standard deviation.*

### CORRELATIONS BETWEEN CENTRAL AND PERIPHERAL MEASURES

No significant relationships were found between PFC activity, H-reflex, and step times. Changes in Oxy-Hb concentrations did not correlate with H-reflex variability and step time variability. Changes in Oxy-Hb concentrations in the left cortex due to dual task and changes in step time showed the highest correlation of 0.420 close toward a trend; *p* = 0.11. Error rates of cognitive task performance were not correlated with significantly higher or lower concentrations of Oxy-Hb or Deoxy-Hb or changes due to single and dual task. Moreover changes in H-reflex variability did not correlate with step time variability.

## DISCUSSION AND CONCLUSION

We found healthy young adults responded to additional cognitive loading during treadmill walking with increased PFC activation, but unlike individuals after stroke or the elderly, this activation was not associated with altered gait parameters (Al-Yahya, 2011). Further, there was no change in the amplitude of the H-reflex during stance in either fast or dual task walking conditions. It was hypothesized that the H-reflex amplitude would reduce during the stance phase of walking when participants were simultaneously performing a cognitive task. However, whilst we observed no change in amplitude of the reflex there was a trend toward increase in H-reflex amplitude variability under both fast and dual task walking conditions. In earlier studies Capaday and Stein (1986),^1987^ found decreases in H-reflex amplitudes from standing to walking and with increasing walking speed throughout the gait cycle, whereas Simonsen and Dyhre-Poulsen (1999), Simonsen et al. (2012) showed increases in H-reflex amplitudes from walking to running. In agreement with these inconsistent results this study confirmed that there is no clear direction in which the H-reflex amplitude is altered, but that an increased walking task difficulty by speed or dual task may increase the variability of the H-reflex amplitude. The absence of changes in gait parameters between different walking conditions indicates that young healthy individuals are able to cope with additional cognitive loads and changes in speed during walking. Therefore it is proposed that the observed increases in PFC activity allowed individuals to perform additional tasks simultaneously, without affecting cortical output onto the measured peripheral reflexes and thus gait control. When exploring correlations between dual task changes in PFC activation and step time, the highest Pearson r^2^ found was 0.420 which was not significant (*p* = 0.11). This indicates that in a healthy young population central mechanisms are activated in response to cognitive loads but that reflex activity and gait performance can successfully be maintained.

Our findings are important as they set out a non-pathological response of reflex control alongside central adaptations to cognitive load in healthy young adults at both self-selected and fast walking speeds. Previous studies (Capaday and Stein, 1986,^1987^; Chalmers and Knutzen, 2000; Ferris et al., 2001) have found both decreases and increases in H-reflex amplitude with increases in walking speed (Simonsen and Dyhre-Poulsen, 1999; Schneider et al., 2000; Simonsen et al., 2012) which suggest that central control mechanisms are involved in H-reflex pathways during activities like walking. The stance phase of gait is important for stability and propulsion during gait and thus of importance to understand mechanisms affecting balance. Our study found a very stable H-reflex during the stance phase of walking within younger subjects. The swing phase of the gait cycle shows a different response which could now be explored in dual task walking conditions. Our findings suggest that central control, measured with prefrontal activation changes, occurred in response to altered walking demands but that these did not affect peripheral reflexes, as measured by the H-reflex through supra-spinal cortical outputs directly controlling motor neuron excitability. Increased impact of cognitive load has been shown during backward walking (Kurz et al., 2012) and during dual tasking in older adults (Seidler et al., 2011). The increase in associated gait decrements in the older populations, particularly those with neurological damage, suggests an age-related shift from automatic to attentional control of movement as walking ability declines (Seidler et al., 2011). Investigation of the impact on both PFC and H-reflex in the older population may elucidate the mechanism behind this behavioral response.

In our younger population, no significant changes in PFC activity were found with increased speed, suggesting there might be differences in control mechanisms of faster speeds, or greater capacity for adaptation in younger population. Importantly peripheral changes were not related to cortical changes. This supports the hypothesis that in healthy individuals there is adequate central capacity to cope with subtle changes in walking and that any peripheral changes may be minimal and separately mediated.

The methodologies used in this study do have some limitations. fNIRS is a developing modality with great opportunities (Belda-Lois et al., 2011), but it also has a poor spatial resolution, low depth penetration and is variable with regards to signal quality between individuals (Toronov et al., 2007; Seraglia et al., 2011). Furthermore due to practical reasons we only measured the PFC, and were limited due to patient comfort in our testing time thus limiting our ability to test the H-reflex throughout the gait cycle and from exploring other motor networks (Suzuki et al., 2004; Kurz et al., 2012; Karim et al., 2013) which may provide further insight into gait and balance control. Our study may be underpowered and prone to type II error. The use of the H-reflex in order to explore walking has inherent practical challenges of using the appropriate intensity of stimulus, timing of the stimulus (Simonsen et al., 2013), protocol (Mynark, 2005), and control of the amount of body weight (Hwang et al., 2011); however, we used techniques with established reliability (Simonsen and Dyhre-Poulsen, 2011; König et al., 2013). Nevertheless the H-reflex has been shown to be effective in exploring the normal response to postural threat, and perhaps by measuring the reflex in mid stance changes at heel strike or other areas of the gait cycle were missed (Krauss and Misiaszek, 2007). Although gait control has not been explored before, differences in the H-reflex during upright stance have been found between the elderly and young in balance responses (Baudry et al., 2010; Baudry and Duchateau, 2012). Considering the high variability in H-reflex and fNIRS between participants, the measures used, although normalized, may not have been sensitive enough to pick up correlations between central and peripheral mechanisms. However, it is important to replicate this research in elderly individuals and neurological populations to explore relationships between the mechanisms in those populations.

We used a treadmill for our study, which is not reflective of an overground walking and normal walking control, since individuals were unable to respond to simultaneous cognitive demand by slowing down (Al-Yahya et al., 2009). Although previous studies using treadmill testing have shown changes in gait parameters, the method may lack some ecological validity for understanding gait control for community mobility. Our population selected an average walking speed of 1.2 ms^-1^ which is lower than the average walking speed for this age group (Bohannon and Williams Andrews, 2011) resulting in fast walking speeds, set at 120% of normal walking speed, which were more reflective of a normal walking pace.

Our results have shown that cognitive load does increase activity in the PFC but this is not associated with a change in H-reflex modulation during stance or gait parameters. Gait control mechanisms under speed and dual task conditions now need to be explored in older adults, and people prone to falls or poor balance and mobility.

## Conflict of Interest Statement

The authors declare that the research was conducted in the absence of any commercial or financial relationships that could be construed as a potential conflict of interest.
